# Metabolomic Signatures of High Red Blood Cell Distribution Width

**DOI:** 10.1093/geroni/igaa057.3330

**Published:** 2020-12-16

**Authors:** Abigail Corkum, Qu Tian, Luigi Ferrucci

**Affiliations:** National Institute of Aging, Baltimore, Maryland, United States

## Abstract

Red blood cell distribution width (RDW) describes the amount of variation in blood cell volume and size and increases with age. Higher RDW predicts all-cause mortality, metabolic syndrome, diabetes, and markers of glycemic control, such as glycosylated hemoglobin. However, mechanisms that connect high RDW with these health outcomes are unknown. Thus, identification of high risk in these patients cannot be addressed. This study aims to identify metabolites and pathways that are associated with high levels of RDW in community-dwelling older adults. Using data from the Baltimore Longitudinal Study of Aging, we identified 1,004 cognitively normal participants (mean age: 67.1±13, 48% women, 26% black) with concurrent data on RDW and comprehensive targeted plasma metabolites by Biocrates p500. Participants were grouped into RDW quartiles (Q1:14%). Associations of metabolites with quartiles of RDW were examined using multivariable linear regression with Q1 being the reference group. Models were adjusted for age, sex, and race. Compared to Q1, Q4 had higher concentrations of SM(OH)C14:1, PC ae C30:2, and hypoxanthine, and lower concentrations of DHEAS, Cortisol, Tryptophan, and Hex2Cer(d/18:1/24:0) (all p<0.01). These metabolites are critical components of sphingolipid metabolism and steroid hormone biosynthesis pathways. Elevated RDW was associated with metabolites derived from classes of hormones, amino acids, ceramides, sphingomyelins, PCs, and nucleobases. Individuals with elevated RDW (i.e. ≥14%) may have disrupted sphingolipid metabolism and steroid hormone biosynthesis. These pathways can be targeted for prevention.

